# The Histone Demethylase JMJD2A Modulates the Induction of Hypertrophy Markers in iPSC-Derived Cardiomyocytes

**DOI:** 10.3389/fgene.2018.00014

**Published:** 2018-02-09

**Authors:** Wendy Rosales, Fernando Lizcano

**Affiliations:** Center of Biomedical Research, Universidad de La Sabana, Chía, Colombia

**Keywords:** hiPSCs, cardiomyocytes, JMJD2A, ET-1, cardiac hypertrophy

## Abstract

The development of cardiovascular pathologies is partly attributed to epigenetic causes, including histone methylation, which appears to be an important marker in hearts that develop cardiac hypertrophy. Previous studies showed that the histone demethylase JMJD2A can regulate the hypertrophic process in murine cardiomyocytes. However, the influence of JMJD2A on cardiac hypertrophy in a human cardiomyocyte model is still poorly understood. In the present study, cardiomyocytes derived from human induced pluripotent stem cells (iPSCs) were used. Hypertrophy was induced by angiotensin II and endothelin-1 (ET-1), and transfections were performed to overexpress JMJD2A and for small interfering RNA (siRNA)-induced silencing of JMJD2A. Gene expression analyses were determined using RT-PCR and Western blot. The expression levels of B-type natriuretic peptide (BNP), natriuretic peptide A (ANP), and beta myosin heavy chain (β-MHC) were increased by nearly 2–10-fold with ET-1 compared with the control. However, a higher level of JMJD2A and UTX was detected, whereas the level of JMJD2C was lower. When cardiomyocytes were transiently transfected with JMJD2A, an increase close to 150% in BNP was observed, and this increase was greater after treatment with ET-1. To verify the specificity of JMJD2A activity, a knockdown was performed by means of siRNA-JMJD2A, which led to a significant reduction in BNP. The involvement of JMJD2A suggests that histone-specific modifications are associated with genes encoding proteins that are actively transcribed during the hypertrophy process. Since BNP is closely related to JMJD2A expression, we suggest that there could be a direct influence of JMJD2A on the expression of BNP. These results may be studied further to reduce cardiac hypertrophy via the regulation of epigenetic modifiers.

## Introduction

At the molecular level, post-translational modifications in histones play an important role in the regulation of gene expression, and they are also part of the epigenetic memory system that regulates cell fate and functional specificity (Gurard-Levin and Almouzni, 2014). Therefore, the molecules that may be involved in the regulation of these processes are promising targets for the development of therapies to treat diseases, including cardiovascular diseases, which are the leading cause of death worldwide (McKenna et al., 2017).

Cardiac hypertrophy is triggered by mechanic-transduction effects and by neurohormonal effects (Shimizu and Minamino, 2016). However, most models of cardiac hypertrophy (deAlmeida et al., 2010) have been realized through mechanical effects through transverse aortic constriction (TAC) (Zhang et al., 2011), and relatively few studies have evaluated neurohormonal activity in the pathogenesis of cardiac hypertrophy (Shimizu and Minamino, 2016). Cardiomyocytes derived from human induced pluripotent stem cells (hiPSCs) offer a model closer to the electrophysiological behavior of the human heart (Drawnel et al., 2014). Angiotensin II (Ang II) and endothelin-1 (ET-1) have been established for a model to promote hypertrophy; the underlying mechanism is related to the induction of several signaling cascades, which include an increase in intracellular calcium Ca^2+^ through the activation of calcium/calmodulin-dependent protein kinase II (CaMKII) or calcineurin, which is an end that mediates cardiac growth (Bupha-Intr et al., 2012).

In biological processes including cardiac diseases, histone demethylation has proven to be an important epigenetic marker; the Jumonji protein family functionally excels in these systems. This family of proteins includes JMJD2A (Gray et al., 2005), the first discovered tri-demethylase specific for the lysine residues H3K9 and H3K36 (Huang et al., 2006; Klose et al., 2006); this protein has recently been shown to be involved in the development of cardiac hypertrophy in a murine model, presenting a possible relationship between the demethylation of H3K9me3 and this pathology (Zhang et al., 2011; Lizcano and Garcia, 2012). Using murine cardiac cell models, we previously observed that JMJD2A could increase hypertrophy markers without any observable influence from the other studied histone demethylases (Rosales et al., 2016).

To understand the behavior of JMJD2A in cardiac hypertrophy, we used hiPSCs-derived cardiomyocytes as an approximate electrophysiological model of the human heart (Takahashi and Yamanaka, 2006). In addition to recapitulating cardiac biology and overcoming the limitations of existing models of primary heart cells, hiPSCs-derived cardiomyocytes also provide a phenotype that is clinically relevant to cardiac hypertrophy, providing a good platform for studying the electrophysiological properties and the discovery of drugs in these conditions (Fine et al., 2013).

Thus, the present study was performed to evaluate the influence of neurohormones, especially Ang II and ET-1, on the production of cardiac hypertrophy markers and the influence of some histone demethylases on this process. We observed that JMJD2A is the only histone demethylase that has a probable direct influence on hypertrophy markers in the hiPSCs-derived cardiomyocytes that were induced in this pathology.

## Materials and Methods

### Cell Culture and Maintenance

Although cardiomyocytes derived from hiPSCs are increasingly available in the market, for this study, we used cardiomyocytes developed and optimized by Cellular Dynamics International (CDI, Madison, WI, United States). These cells employ episomal reprogramming that uses circular DNA vectors to deliver the pluripotency genes to adult cells; the patented methods focus on the production of differentiated tissue cells in industrial quantities of high quality and purity, providing cells that demonstrate the phenotypic, electrophysiological and functional characteristics of mature cardiomyocytes (Traister et al., 2014). Cellular dynamics international classifies these cells as Biosafety Level 1 (BSL1) based on the United States Centers for Disease Control and Prevention (Ma et al., 2011).

The hiPSCs-derived cardiomyocytes were plated and maintained according to the guidelines of the manufacturer. The cells were plated on 10 μg/mL fibronectin medium (Invitrogen 33016-015, Carlsbad, CA, United States) as the support matrix for 48 h at a temperature of 37°C and 5% CO_2_. The maintenance medium was changed every 2 days.

### Hypertrophy Induction

To induce a hypertrophic response, cardiomyocytes in the maintenance medium were washed with PBS (Life Technologies 14190, Carlsbad, CA, United States). The medium was changed to Williams’ medium (Life Technologies A12176, Carlsbad, CA, United States) supplemented with Cocktail B (Life Technologies CM4000, Carlsbad, CA, United States) (SWE) with the subsequent addition of 200 μM Ang II (Sigma–Aldrich A9525, San Luis, MO, United States) and 100 μM ET-1 (Sigma–Aldrich E7764, San Luis, MO, United States) at 24 h post-induction initially, except when transfection or silencing was performed prior to the addition of ET-1, which was added 18 h prior to gene or protein analysis.

### Transfection

For transfection studies was used Lipofectamine^®^ 3000 (Thermo Fisher L3000001, Waltham, MA, United States), one of the best method for transfection on this kind of cells. We follow the recommendation of the manufacturer and efficiency of 30 to 40% was expected and the experiments were performed by triplicated. In brief, Cardiomyocytes maintained in 12-well plates were washed with PBS, and the medium was changed to SWE for transfection. The transfection mixture was prepared in 375 μL of Opti-MEM^®^ medium (Thermo Fisher 31985070, Waltham, MA, United States) using 3.75 μL of Lipofectamine^®^ 3000, 5 μL of P3000 Reagent and 2.5 μg of DNA per well. After 20 min at room temperature, the mixture was added to the cells in the wells with SWE medium. At 6 h post-transfection, the medium with the mixture was changed to only SWE medium. Cells were analyzed for changes in molecular expression after transfection of pcDNA-JMJD2A and were compared to cells with an empty plasmid for the control.

### Silencing

Synthetic small interfering RNA (siRNA) oligonucleotides were purchased from Invitrogen (**Table 1**) Different sequences were tested to determine which of the oligonucleotides used was the most efficient for decreasing/silencing the expression of JMJD2A.

**Table 1:** siRNA used to silence JMJD2A.

Reference	Direction 5′ → 3′	Direction 3′ → 5′
*siRNA-JMJD2A (1)*	*AAGUUGACUUCAUAGAAGGUCUCGG*	*CCGAGACCUUCUAUGAAGUCAACUU*
*siRNA-JMJD2A (2)*	*GCCCUAGAGGAGGACUGCUGUUUAU*	*AUAAACAGCAGUCCUCCUCUAGGGC*


Cardiomyocytes were transfected into Nunc^®^ 6-well plates (Thermo Fisher 140685, Waltham, MA, United States). The transfection mixture was prepared in 300 μL of Opti-MEM^®^ medium using 9 μL of Lipofectamine^®^ RNAiMAX (Thermo Fisher 13778100, Waltham, MA, United States), with 100 μM siRNA per well. After 30 min at room temperature, the mixture was added to the cells cultured in maintenance medium. Cells were analyzed after 24 h according to the molecular changes caused by the transfection of the oligonucleotides and compared to a control of Lipofectamine^®^ RNAiMAX without oligonucleotides.

### RT-PCR

High Pure RNA Isolation Kit (Roche 11828665001, Basel, Switzerland) was used to isolate the RNA, which was verified on a 1.5% agarose gel with SYBR^®^ Safe (Thermo Fisher S33102, Waltham, MA, United States) and quantified by means of a NanoDrop. The cDNA was obtained using a Transcriptor First Strand cDNA Synthesis Kit (Roche 04379012001, Basel, Switzerland) and oligo (dT) primers. To analyze the relative levels of mRNA through RT-PCR, FastStart Essential DNA Green Master (Roche 06402712001, Basel, Switzerland) was used. Primers were designed for B-type natriuretic peptide (BNP), natriuretic peptide A (ANP), and beta myosin heavy chain (β-MHC), which are hypertrophy markers, and for lysine demethylase 4A (JMJD2A/KDM4A), lysine demethylase 4C (JMJD2C/KDM4C), and lysine demethylase 6A (UTX/KDM6A), which are histone demethylases, according to the object of the study (**Table 2**). The obtained data were normalized to GAPDH and analyzed with the ΔΔCt method.

**Table 2:** Primers used in RT-PCR.

Primer	Sequence 5′ → 3′	Sequence 3′ → 5′
JMJD2A	*GCCGCTAGAAGTTTCAGTGAG*	*GCGTCCCTTGGACTTCTTATT*
BNP	*TCAGCCTCGGACTTGGAAAC*	*CTTCCAGACACCTGTGGGAC*
ANP	*GACAGACTGCAAGAGGCTCC*	*GCTGCAGCTTAGATGGGATGA*
β-MHC	*CTGTCCAAGTTCCGCAAGGT*	*ATTCAAGCCCTTCGTGCCAA*
JMJD2C	*GCGGTCCCAGAAGTTCGATT*	*TCTAGATTCCCAGCCTTCCCA*
UTX	*CTCCATGGCTAGGACTGCAA*	*AGACACCTAACAGCACTGCC*
GAPDH	*ACCCACTCCTCCACCTTTGAC*	*TGTTGCTGTAGCCAAATTCGTT*


### Western Blot

Once the respective treatments had remained in the medium for the specified length of time, the cells were lysed using RIPA buffer (Abcam 156034, Cambridge, United Kingdom) with the addition of a cocktail of protease inhibitors (Roche 11836170001, Basel, Switzerland) to recover the protein extract for subsequent immunoblotting. After centrifuging the lysate, 200 μg of total proteins was mixed with an equal volume of Sample Buffer (BioRad 161-0737, Hercules, CA, United States), denatured at 97.5°C for 2 min, separated by 12.5% SDS-PAGE with a constant voltage of 120 V and then transferred to a PVDF membrane (EMD Millipore IPVH00010, Billerica, MA, United States). The membrane was incubated in blocking buffer (TBS, 0.1% Tween-20 and 5% skim milk powder) for 1 hour at room temperature and stirred to block non-specific binding. The membrane was then incubated overnight at 4°C with stirring in a 0.1% v/v TBS-Tween-20 solution (Abcam ab128987, Cambridge, United Kingdom) with primary antibodies against JMJD2A (1:2000, Abcam 70786, Cambridge, United Kingdom) and BNP (1:500, Abcam 19645, Cambridge, United Kingdom), washed and incubated with HRP goat anti-rabbit secondary antibody (1:3000, Abcam 6721, Cambridge, United Kingdom) for 1 h at room temperature in 0.1% TBS-Tween-20 solution. Finally, the membrane was exposed for 5 min at room temperature to Luminata-HPR (Millipore WBLUR0500, Billerica, MA, United States) to be developed and visualized by chemiluminescence by means of MyECL equipment (Thermo Fisher, Waltham, MA, United States).

### Statistical Analysis

A one-way analysis of variance (ANOVA) was performed, the assumptions of homogeneity of variance were evaluated through Levene’s test, and the normality of the distribution of residuals was evaluated through the Shapiro–Wilk test; in both cases, the assumptions were validated for all evaluated variables. Factors were reported with significant effects when values of *p* < 0.05 were found. In cases where there were significant effects of the evaluated factors, a comparison of means between the different treatments was performed through the least significant difference (LSD) test. For the data analysis, the SAS 9.4 program was used.

## Results

**FIGURE 1:**
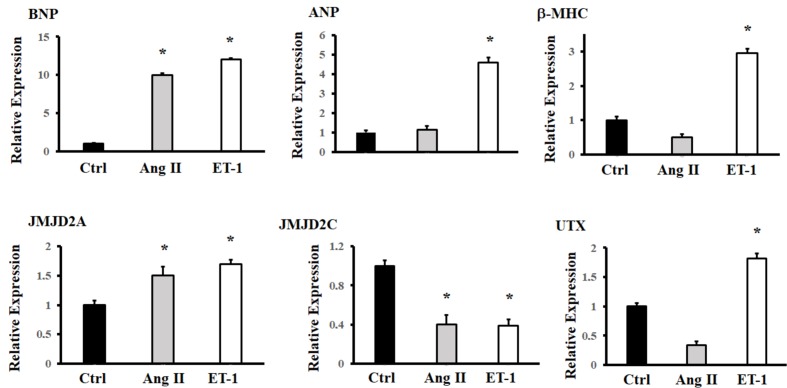
Expression of histone demethylases (JMJD2A, JMJD2C and UTX) and hypertrophy markers (BNP, ANP, and β-MHC) in hiPSCs-derived cardiomyocytes following the induction of hypertrophy with Ang II and ET-1. Analyzed by RT-PCR. ^∗^Means with the same letter are statistically equal under the LSD test with *p* = 0.05.

For the robustness of the technology using cardiomyocytes derived from hiPSCs, sarcomeric organization was not thoroughly investigated. However, Supplementary Figure S1. show a slight increase in the size of cells after the induction of hypertrophy with ET-1. The expression levels of genes that usually increase with the induction of hypertrophy were evaluated; compared to the levels for the controls, the BNP, ANP, and β-MHC levels were higher when the Ang II and ET-1 inducers were used. There was a significant difference in the expression level of the histone demethylases: JMJD2A and UTX expression shown tendency to increase, while JMJD2C expression tended to decrease. In contrast, the UTX level increased significantly only when ET-1 was used. There was a greater difference in gene behavior when ET-1 was used compared to when Ang II was used (**Figure 1**).

**FIGURE 2:**
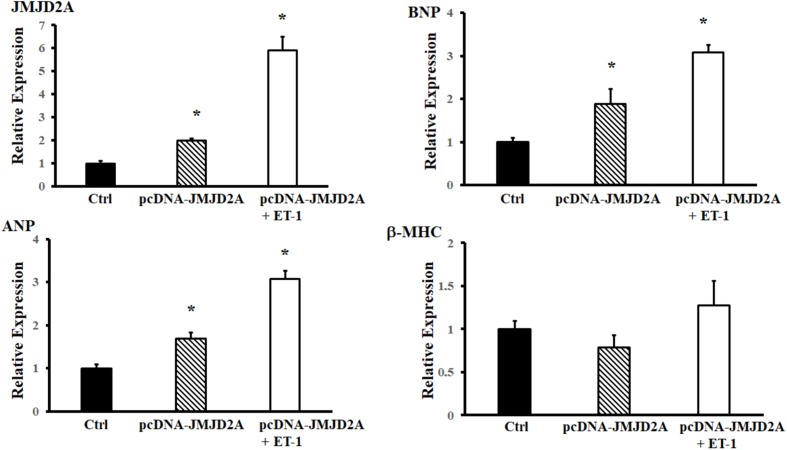
Effect of overexpression of JMJD2A. hiPSCs-derived cardiomyocytes were transiently transfected with pcDNA-JMJD2A or pc-DNA by means of Lipofectamine^®^ 3000 (materials and methods). ET-1 was used to induce hypertrophy in the cells. Analyzed by RT-PCR. ^∗^Means with the same letter are statistically equal under the LSD test with *p* = 0.05.

Considering the behavior of cardiomyocytes when hypertrophy is induced with neurohormonal factors, the next step was to determine whether overexpression of JMJD2A might influence the increase in hypertrophy markers. Different DNA:Lipofectamine ratios from 1:2 to 1:5 in cardiomyocytes cell line was testing for optimize the transfection without affect the cell viability (Supplementary Figure S2). Overexpression of JMJD2A produced an increase in hypertrophy markers; when ET-1 was used as an inducer, this demethylase markedly increased the expression relative to that in the controls. This increase was comparable for the BNP and ANP hypertrophy markers, which showed a significant difference with respect to the controls in both transfection and induction; in contrast, β-MHC did not show a significant difference (**Figure 2**).

**FIGURE 3:**
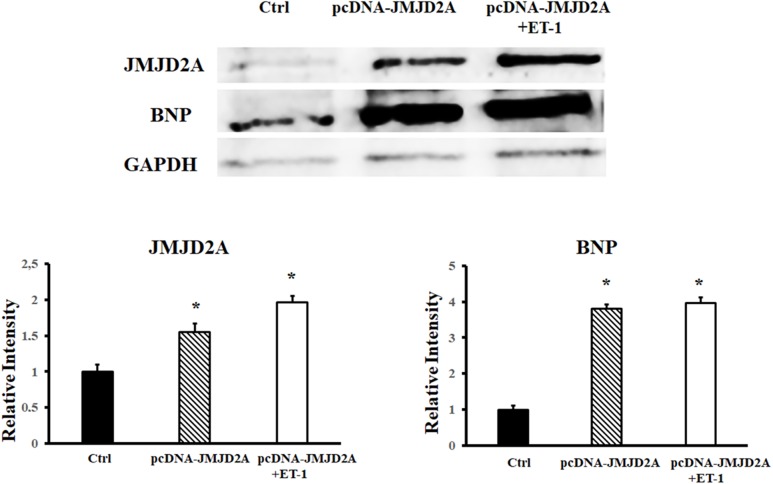
Increase in BNP protein levels after overexpression of JMJD2A. hiPSCs-derived cardiomyocytes were transiently transfected with pcDNA-JMJD2A or pc-DNA by means of Lipofectamine^®^ 3000 (materials and methods). ET-1 was used to induce hypertrophy in the cells. Protein extracts were obtained, and BNP expression was analyzed by Western blotting. ^∗^Means with the same letter are statistically equal under the LSD test with *p* = 0.05.

The expression of hypertrophy markers was confirmed by the observed increase in the expression of the BNP protein after the overexpression of JMJD2A in the cardiomyocytes (**Figure 3**).

**FIGURE 4:**
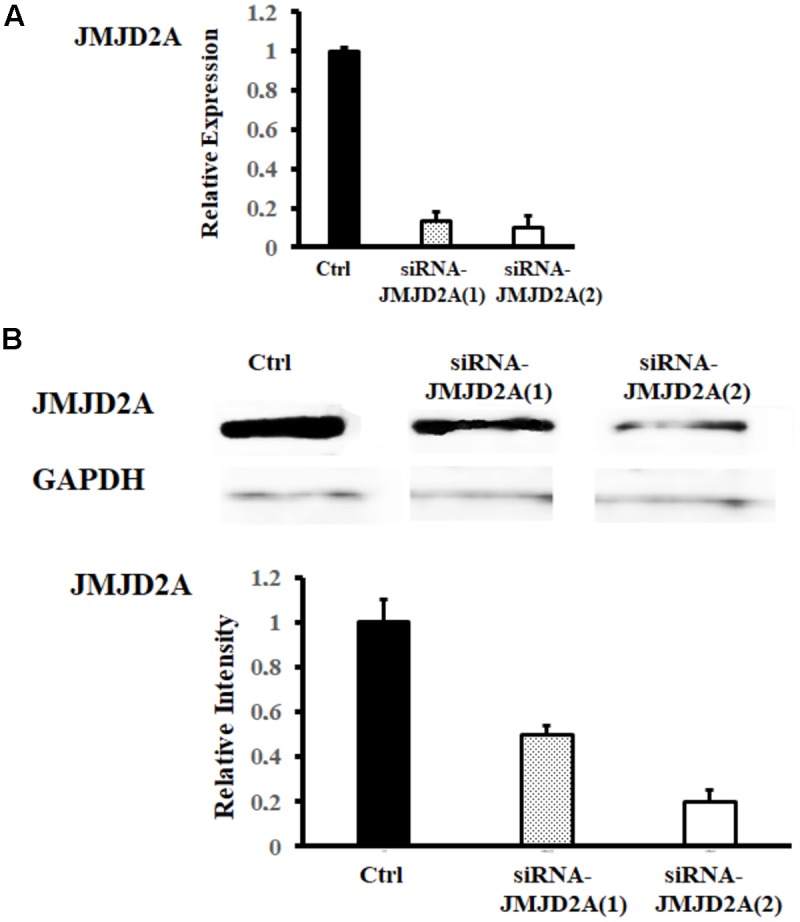
JMJD2A silencing. hiPSCs-derived cardiomyocytes were transfected by means of Lipofectamine^®^ RNAiMAX with oligonucleotides synthesized by Invitrogen. JMJD2A expression was analyzed by RT-PCR and Western blot. ^∗^Means with the same letter are statistically equal under the LSD test with *p* = 0.05. **(A)** We observed a reduction in mRNA of JMJD2A after siRNA-JMJD2A. **(B)** Protein expression of JMJD2A is reduced after siRNA-JMJD2A. The reduction is more intense with siRNA-JMJD2A (2).

To determine the specificity of JMJD2A activity on the expression of hypertrophy markers, we evaluated whether a reduction in JMJD2A could influence the expression of these hypertrophy markers. Two siRNA-JMJD2A molecules were used to decrease the expression of JMJD2A. We found that siRNA-JMJD2A (2) presented better silencing efficiency by significantly decreasing the presence of JMJD2A at both the RNA and protein levels (**Figure 4**).

**FIGURE 5:**
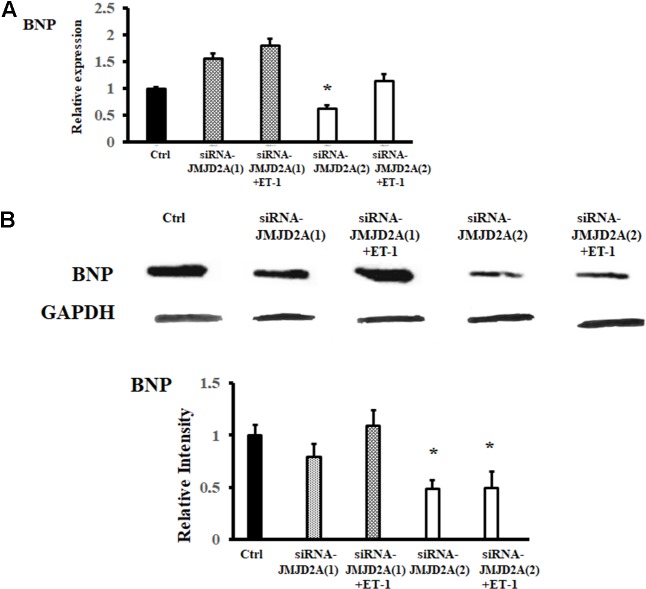
Effect of JMJD2A silencing on BNP expression. hiPSCs-derived cardiomyocytes were transfected by means of Lipofectamine^®^ RNAiMAX. ET-1 was used to induce hypertrophy in the cells, and BNP expression was analyzed by RT-PCR and Western blot. ^∗^Means with the same letter are statistically equal under the LSD test with *p* = 0.05. **(A)** We observed a reduction of BNP mRNA after the transfection of siRNA-JMJD2A (2). BNP mRNA expression is partially recover by ET-1. **(B)** BNP protein expression is reduced after the transfection of siRNA-JMJD2A (2).

To determine whether the expression of hypertrophy markers was altered in the absence of JMJD2A but in the presence of hypertrophy inducers, siRNAs were used with subsequent addition of ET-1. The results showed that the BNP expression level relative to the control level did not increase significantly when ET-1 treated siRNA-JMJD2A (2) was used (**Figure 5**).

## Discussion

In the present work, we observed that epigenetic modifications may exert an important effect on the expression of hypertrophy markers in hiPSCs-derived cardiomyocytes. The histone demethylase JMJD2A, can induce the expression of these markers in human cardiac cells.

Some studies have described the influence of JMJD2A on the hypertrophic process of the heart (Zhang et al., 2011; Hohl et al., 2013). However, these studies have been based on mechanic-transduction mechanisms (TAC) in murine models (Traister et al., 2014). In the present study, we evaluated the neurohormonal effect of ET-1 in human cardiomyocytes, observing the relevant role of JMJD2A in the modulation of cardiac hypertrophy markers. Cardiomyocytes derived from human iPSCs are well documented model of cardiac hypertrophic. However, despite lack of increase in cell size after use ET-1 observed by some authors, they did see downstream changes in hypertrophic protein profiles in treated cells (Hong et al., 2011). On the other hand, both ET-1 and Ang II (Angiotensin II) produced only small changes in cell size, but robust increases in ANP and BNP (Natriuretic Peptides) in hiPSC-CMs (Foldes et al., 2014). In the Supplementary Figure S1 we observed a light increase in the size of cardiomyocytes after the treatment with ET-1.

The literature on physiological studies of the function of signaling pathways, apoptosis and kinases, among others, is extensive (Zhi et al., 2012; Vanezis et al., 2016); because of their relevance and reactivity, in addition to their wide implementation in different studies, Ang II and ET-1 were used in this study.

The overexpression of JMJD2A in the hiPSCs-derived cardiomyocytes by transient transfections demonstrated a good physiological behavior, which allowed us to consistently evaluate the effect of JMJD2A on several cardiac hypertrophy markers in these cardiomyocytes (Aggarwal et al., 2014).

However, one of the limitation of transient transfection studies in these kind of cells is the fact that they cannot proliferate, and the efficiency of transfections is around 30 to 40% with lipofectamine 3000. For this reason, we performed the experiments for triplicate in order to normalize the results after JMJD2A transfection. JMJD2A is a member of a JMJD family that catalyze the demethylation reaction of trimethylated H3K9 and H3K36. This function of JMJD2A can increase the expression of some genes by means of an interaction with transcription factors (Hohl et al., 2013).

Because cardiac hypertrophy is observed in many conditions, including myocardial infarction, ischemia, hypertension, valvular dysfunction and toxic side effects caused by stimulation through small molecules/proteins, there is a need for reproducible methods for the detection, quantification and eventual treatment of this condition (Zhi et al., 2012). Among histone demethylases, both JMJD2A and UTX tend to increase, whereas JMJD2C behaves inversely when hypertrophy inducers are used (**Figure 1**). For the hypertrophy markers, we observed that the expression levels of both BNP and ANP increase, as indicated by RT-PCR, under the influence of ET-1. In cardiac hypertrophy processes, an increase in β-MHC is usually observed, as in our study, with a reduction in α-MHC (Cox and Marsh, 2014).

Although JMJD2A and JMJD2C are histone demethylases for H3K9 and H3K36 belonging to the same protein family (Lizcano and Garcia, 2012), our study demonstrates that JMJD2A and JMJD2C have opposite expression patterns when cardiac hypertrophy is induced. JMJD2C plays a role in embryonic cells both in the renewal of pluripotency itself and as a regulator of embryonic cell differentiation (Das et al., 2014; Tomaz et al., 2017). We observed a reduction in the differentiation capacity of pluripotent mesenchymal adipose cells after JMJD2C overexpression in human subcutaneous adipose tissue (Lizcano et al., 2011). Our results indicate that the role of JMJD2C in the development of cardiac hypertrophy is likely limited.

In contrast, UTX, like JMJD3, favors H3K27 a demethylase function, which is important for the process of cardiac cell differentiation from mesodermal precursors (Lee et al., 2012; Welstead et al., 2012). In our model, UTX did not increase significantly during Ang II-induced hypertrophy, probably because this demethylase for H3K27 plays an important role in the differentiation of cardiac cells from progenitor cells, suggesting that the signaling pathway associated with UTX functioning may vary when ET-1 is used as a hypertrophy inducer (Jiang et al., 2013).

Regarding the overexpression of JMJD2A using the hiPSCs-derived cardiomyocytes as a cellular model and the subsequent induction of hypertrophy using ET-1, the expression levels of BNP increased as described in the results. The use of small molecules that induce a pathology in this type of model provides an overview of how useful these types of cells derived from hiPSCs can be for studies of drug cardiotoxicity as a preclinical control or for the process of discovering drugs and identifying the harmful effects that occur when a large amount of chemical substrate is available (Rana et al., 2012).

The silencing of JMJD2A expression by siRNA (**Figure 4**) affected BNP expression in the hiPSCs-derived cardiomyocytes. This reduction demonstrates a direct effect of JMJD2A on cardiac hypertrophy markers. The reduction in BNP levels observed by means of RT-PCR and Western blot (**Figure 5**) may occur because the promoter region of BNP is methylated; this region is specifically located in H3K9 (Hohl et al., 2013). One of the most interesting observations in this work is that the expression of BNP, a marker of hypertrophy, does not significantly increase after induction with ET-1 in the absence of JMJD2A, which could be an adaptation of the cardiomyocytes to this type of stimulus.

## Conclusion

JMJD2A is so far, the only identified histone demethylase that can induce hypertrophy markers in cardiac cells that were differentiated from induced pluripotent human cells (hiPSCs). This effect is likely directly mediated by the demethylase activity in the BNP and ANP regulatory region, among other fetal cardiac genes. These observations may be of great importance for a possible therapeutic target for reducing pathologic cardiac hypertrophy.

## Author Contributions

WR and FL conceived of the presented idea, designed the analysis, developed the theory and performed the analysis. WR carried out the experiment. FL verified the analytical methods and the data. FL encouraged WR to investigate and supervised the finding of this work. All the authors discussed the results and contributed to write the paper.

## Conflict of Interest Statement

The authors declare that the research was conducted in the absence of any commercial or financial relationships that could be construed as a potential conflict of interest. The reviewer AA and handling Editor declared their shared affiliation.
