# Supplementary material for: The Histone Demethylase JMJD2A Modulates the Induction of Hypertrophy Markers in iPSC-Derived Cardiomyocytes
Source: Front Genet. 2018 Feb 9;9:14. doi: 10.3389/fgene.2018.00014 (PMC5811633; doi:10.3389/fgene.2018.00014)
Supplement: Supplementary file 1 [file Data_Sheet_1.DOCX]

Supplementary Material

The histone demethylase JMJD2A modulates the induction of hypertrophy markers in iPSC-derived cardiomyocytes

Wendy Rosales^1^ , Fernando Lizcano*^1^

^1^ Center of Biomedical Research University of La Sabana (CIBUS), University of La Sabana, Chía, Colombia.

*** Correspondence:**Fernando Lizcano
[fernando.lizcano@unisabana.edu.co](mailto:fernando.lizcano@unisabana.edu.co)

# Supplementary Figures
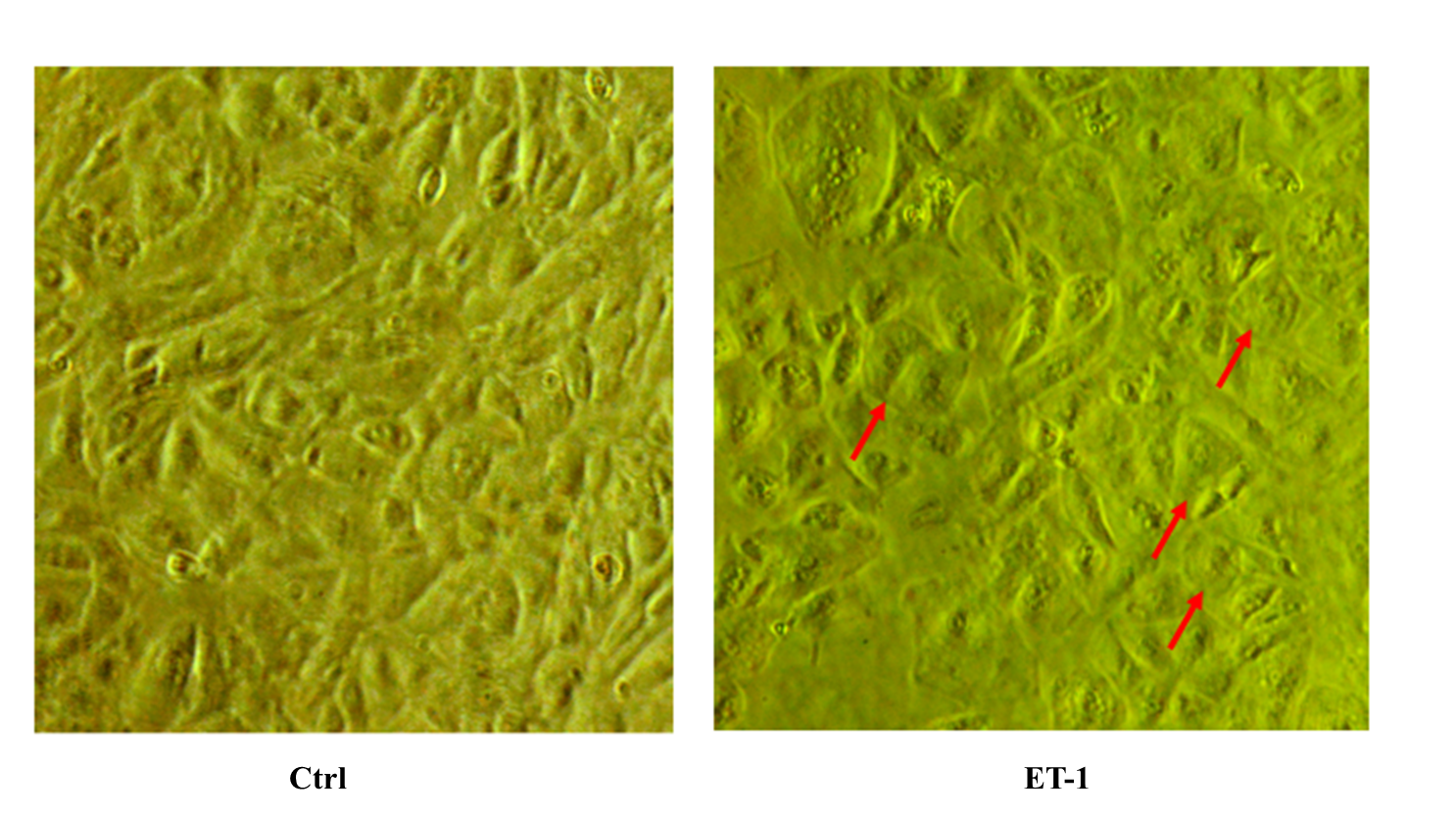


**Figure S1.** Cardiomyocytes derived from hiPSC before and after the induction of hypertrophy with Endothelin 1. In the figure we can observe a slight increase in the size of cells after the induction of hypertrophy with endothelin 1 (ET-1).


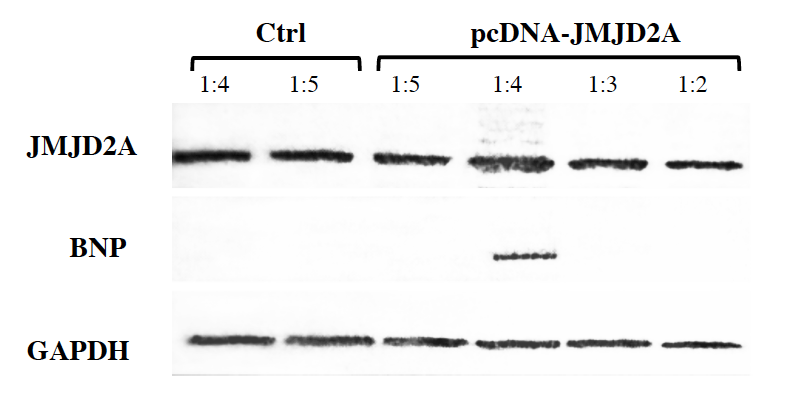


**Figure S2.** In order optimize the transfection without affect the cell viability. First was testing different DNA:Lipofectamine 3000 ratios from 1:2 to 1:5 in cardiomyocytes. The best result was 1:4 DNA:Lipofectamine 3000 ratio compared with the others relations like evidence the membrane.
